# Serial evaluation of serum thymidine kinase activity is prognostic in women with newly diagnosed metastatic breast cancer

**DOI:** 10.1038/s41598-020-61416-1

**Published:** 2020-03-11

**Authors:** Anna-Maria Larsson, Pär-Ola Bendahl, Kristina Aaltonen, Sara Jansson, Carina Forsare, Mattias Bergqvist, Charlotte Levin Tykjær Jørgensen, Lisa Rydén

**Affiliations:** 1 Department of Clinical Sciences, Lund, Division of Oncology and Pathology, Lund University, Lund, Sweden; 2 Department of Hematology, Oncology and Radiation Physics, Skåne University Hospital, Lund, Sweden; 3 Department of Laboratory Medicine, Division of Translational Cancer Research, Lund University, Lund, Sweden; 4 Biovica, Uppsala, Sweden; 5 Department of Clinical Sciences, Lund, Division of Surgery, Lund University, Lund, Sweden; 6 Department of Surgery, Skåne University Hospital, Lund, Sweden

**Keywords:** Breast cancer, Tumour biomarkers

## Abstract

The rapid development of new therapies in metastatic breast cancer (MBC), entails a need for improved prognostic and monitoring tools. Thymidine kinase 1 (TK1) is involved in DNA synthesis and its activity correlates to outcome in cancer patients. The aim of this study was to evaluate serum TK1 activity (sTK1) levels in MBC patients as a tool for prognostication and treatment monitoring. 142 women with MBC scheduled for 1^st^ line systemic treatment were included in a prospective observational study. sTK1 was measured at baseline (BL) and at 1, 3 and 6 months and correlations to progression-free and overall survival (PFS, OS) evaluated. High sTK1 levels (above median) correlated to worse PFS and OS at BL, also after adjusting for other prognostic factors. sTK1 levels were significantly associated with PFS and OS measured from follow-up time points during therapy. Changes from 3 to 6 months during therapy significantly correlated to PFS and OS, whereas early changes did not. We could demonstrate sTK1 level as an independent prognostic factor in patients with newly diagnosed MBC. Changes in sTK1 levels from 3 to 6 months correlated to PFS and OS. Future studies of sTK1 are warranted to further define its clinical utility.

## Introduction

Breast cancer is the most common malignant disease in women and although the 5-year survival is approaching 90%, 20–30% of women with initially local disease will develop metastatic disease^1^. Even though treatment regimens have improved, the median survival in women with metastatic breast cancer (MBC) is approximately 2 years and the 5-year survival is only 25%^2^. MBC is generally an incurable disease and therapy is focused on symptom palliation, extending survival and improving quality of life^2^. Monitoring treatment response in MBC remains a clinical challenge and current guidelines suggest a combination of imaging, clinical assessment and in addition application of serum tumor markers if initially elevated^2–4^. Imaging is golden standard for tumor response evaluations in patients with measurable disease^5^. However, studies report that 10–40% of MBC patients have non-measurable disease^6,7^. There is a clinical need for better tools to improve prognostication and therapy monitoring in MBC.

Blood-borne markers are gaining attention since they carry real-time information on tumor progression and are easily evaluated in a simple blood test. Thymidine Kinase 1 (TK1) has been proposed as a marker of cell proliferation. The main function of TK1 is involvement in nucleotide metabolism with a fundamental role in DNA synthesis, essential for cell proliferation^8–10^. In patients with different tumor types, TK1 activity has been shown to carry prognostic information and potential in tumor monitoring^11–14^. In breast cancer, high TK1 activity in blood has been associated with worse prognosis^15^. Serum TK1 activity (sTK1) level has been proposed as a prognostic marker in MBC patients during endocrine therapy (ET) and as an indicator for early response in these patients^16,17^. Furthermore, it has been suggested as a marker for therapy response in cyclin-dependent kinase 4/6 inhibition (CDK-I) in patients with early breast cancer treated with neoadjuvant palbociclib^18^. However, little is known about the potential prognostic role of sTK1 levels during systemic therapy in patients with newly diagnosed MBC, previously untreated for metastatic disease.

This study aims to evaluate sTK1 levels in women with MBC scheduled for 1^st^ line systemic therapy (ET, ChT or HER2 targeted therapy), at BL and by serial sampling during therapy, to further investigate the potential role of sTK1 in prognostication and therapy monitoring of MBC.

## Results

### Patient characteristics

Median age of patients at study inclusion was 65 (range 40–90) years. Twenty nine patients (20%) were diagnosed with *de novo* MBC and 113 patients (80%) were diagnosed with distant recurrence. Median metastasis-free interval was 4.6 years (range 0–37). Forty three patients (30%) had more than three metastatic loci and 83 patients (58%) had visceral metastasis at BL. The majority of patients, 99 (70%), had estrogen receptor positive (ER+) disease whereas 15 patients (11%) had HER2 positive disease and 25 patients (18%) had triple negative breast cancer (TNBC). Subtype was determined primarily from assessment of biopsies from metastatic lesions and secondarily from the primary tumor. Fifty-seven patients (40%) received endocrine therapy (ET), 64 patients (45%) received chemotherapy (ChT) and 13 patients (9%) received HER2-directed therapy in combination with ET or ChT as 1^st^ line therapy. For the remaining eight patients (6%) systemic therapy was not initiated or terminated early and patients received best supportive care. The median follow-up time was 25 months (range 7–69 months) for patients alive at last registered health contact.

### sTK1 levels in MBC patients

**Figure 1:**
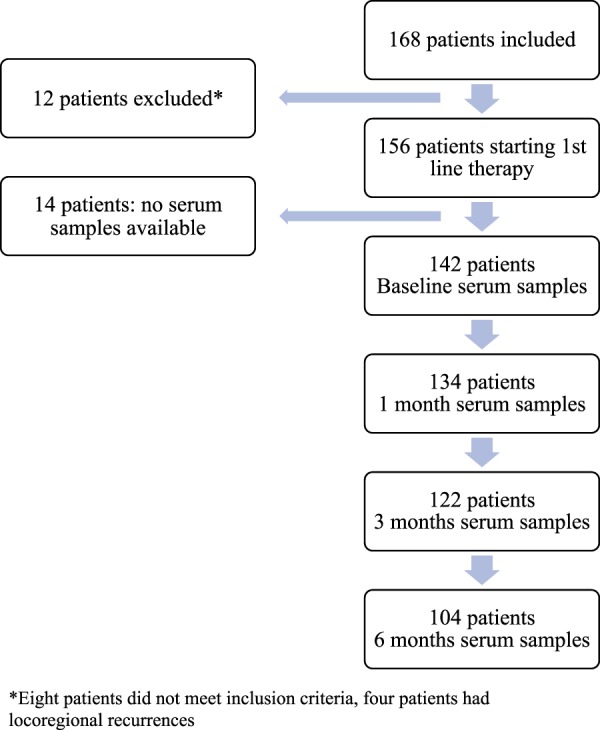
Flowchart of study cohort and time points for serum sampling.

sTK1 levels were measured at BL for 142 patients and at 1, 3 and 6 month for 134, 122 and 104 patients respectively (see study flow chart, Fig. 1).

**Figure 2:**
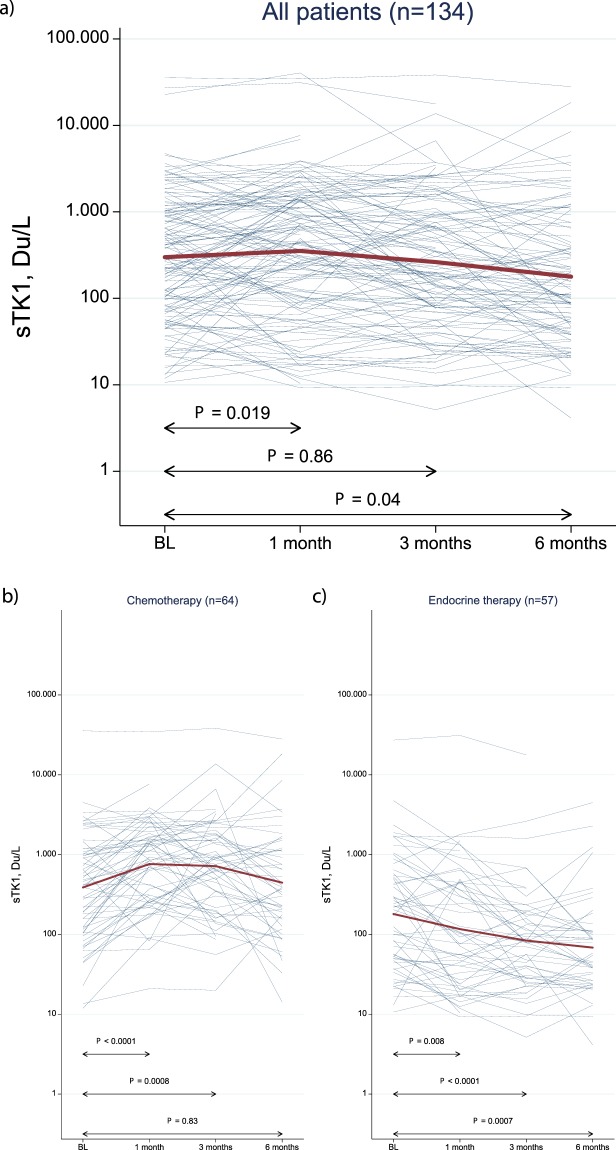
Changes in sTK1 activity levels during systemic therapy. Changes in log sTK1 levels from baseline to 1, 3 and 6 months during systemic therapy for all patients (**a**) and for subgroups based on treatment regimen; chemotherapy (**b**) versus endocrine therapy (**c**). Red lines represent the median sTK1 levels in the respective groups.

The median sTK1 level at baseline (BL) was 391 Du/L (range: 10–35520 Du/L) and median levels were reduced during systemic treatment (Fig. 2, Table S1). When evaluating changes in sTK1 levels during treatment, patients were divided into three groups based on type of therapy; ChT, ET and HER-2 directed therapy (in combination with ChT or ET). The group of patients receiving ET had lower sTK1 levels at BL (median sTK1: 204 Du/L, range: 11–27230 Du/L) (Fig. 2c, Table S1) whereas patients receiving ChT had a median sTK1 level of 420 Du/L (range: 12–35520 Du/L) at BL (Fig. 2b, Table S1). Patients receiving HER2-directed therapy had the highest BL median sTK1 level of 1037 Du/L (range 16–22740 Du/L; Table S1). During treatment, sTK1 levels showed different dynamics in the various therapy groups. In patients receiving ChT there was a significant increase in sTK1 levels from median 420 Du/L at BL to median 874 Du/L (range: 21–34510 Du/L, *P* < 0.001) at 1 month. After 3 and 6 months of ChT, sTK1 levels were reduced to median 759 Du/L (range: 20–38310 Du/L) and median 387 Du/L (range: 14–28030) respectively (Fig. 2b, Table S1). In ET treated patients, sTK1 levels were significantly reduced after 1 month of therapy (*P* = 0.008), and continuously reduced at 3 and 6 months respectively (Fig. 2c, Table S1). As for patients treated with HER-2 directed therapy, the initial median level was 1037 Du/L at BL and sTK-1 levels were reduced during treatment to 913 Du/L at 1 month, 197 Du/L at 3 months and 107 Du/L at 6 months, respectively (Table S1). When comparing the sTK1 distributions at baseline in chemotherapy treated patients stratified for subtype (ER+ versus TNBC) using Mann Whitney U-test, the evidence for a difference was very weak (P = 0.52).

### sTK1 levels in correlation to clinicopathological variables in MBC

To evaluate sTK1 levels in relation to important clinicopathological factors, patients were divided into groups with high versus low sTK1 levels, based on median sTK1 levels at BL (Table 1). We found that high sTK1 levels correlated significantly to worse performance status, high number of metastatic loci (≥3) and type of therapy (ChT and HER2-directed therapy). Furthermore, there was a strong correlation between sTK1 levels and number of circulating tumor cells (CTC) where the majority of patients with high CTC count (≥5 cells/7.5 ml of blood) also had high sTK1 levels (*P* < 0.001) (Table 1). In addition, there was suggestive evidence for a correlation between high sTK1 level and high Ki67 expression in biopsies from metastatic lesions (*P* = 0.06) (Table 1).

**Table 1:** Clinicopathological variables in MBC patients at baseline, stratified for sTK1 activity levels (high/low).

	All patients, N = 142 (%)	BL sTK1 levels Low (<median) N = 71 (%)	BL sTK1 levels High (≥median) N = 71 (%)	*P*-value
**Age** at MBC diagnosis (median, range)	65 (40–90)	66 (40–90)	65 (41–84)	0.76^a^
**Metastasis-free interval (years)**				
0	29 (20)	12 (17)	17 (24)	0.15^b^
>0–3	26 (18)	11 (15)	15 (21)	
>3	87 (61)	48 (68)	39 (55)	
**BL ECOG**				
0	80 (58)	49 (71)	31 (45)	**0.001**^**b**^
1	36 (26)	15 (22)	21 (30)	
2	22 (16)	5 (7)	17 (25)	
Unknown	4	2	2	
**NHG**				
I	12 (11)	8 (13)	4 (8)	0.15^b^
II	59 (52)	33 (55)	26 (49)	
III	42 (37)	19 (32)	23 (43)	
Unknown	29	11	18	
**T (size)**				
T1	51 (38)	28 (41)	23 (35)	0.37^b^
T2	47 (35)	22 (32)	25 (38)	
T3	18 (14)	12 (18)	6 (9)	
T4	17 (13)	6 (9)	11 (17)	
Unknown	9	3	6	
**Node**				
Neg	41 (34)	24 (36)	17 (30)	0.48^c^
Pos	81 (66)	42 (64)	39 (70)	
Unknown	20	5	15	
**Breast cancer subtype**^**a**^				
ER+ HER2−	99 (71)	54 (77)	45 (65)	0.30^c^
HER2+	15 (11)	6 (9)	9 (31)	
ER− HER2−	25 (18)	10 (14)	15 (22)	
Unknown	3	1	2	
**No of metastatic sites**				
<3	100 (70)	56 (79)	44 (62)	**0.03**^**c**^
≥3	42 (30)	15 (21)	27 (38)	
**Visceral metastasis**				
No	59 (42)	31 (44)	28 (39)	0.61^c^
Yes	83 (58)	40 (56)	43 (61)	
**Treatment**				
Chemotherapy	64 (48)	30 (42)	34 (54)	**0.03**^**c**^
Endocrine therapy	57 (42)	37 (52)	20 (32)	
HER2-directed therapy	13 (10)	4 (6)	9 (14)	
Unknown	8	0	8	
**Ki67% metastasis**				
Low (<20)	24 (33)	16 (43)	8 (22)	0.06^c^
High (≥20)	49 (67)	21 (57)	28 (78)	
Unknown	69	34	35	
**CTC count**				
<5	67 (48)	50 (70)	17 (25)	**<0.001**^**c**^
≥5	73 (52)	21 (30)	52 (75)	
Unknown	2	0	2	

*Abbreviations:* MBC, metastatic breast cancer; BL, baseline; ECOG, Eastern Cooperative Oncology Group; NHG, Nottingham Histological Grade; ER, estrogen receptor; HER2, human epidermal growth factor receptor 2; No, number; CTC; circulating tumor cell.

^a^*P*-value from Mann Whitney’s test.

^b^*P*-value from Pearsons Chi-Square test for trend.

^c^*P*-value from Pearsons Chi-Square test.

### sTK1 levels and survival

**Figure 3:**
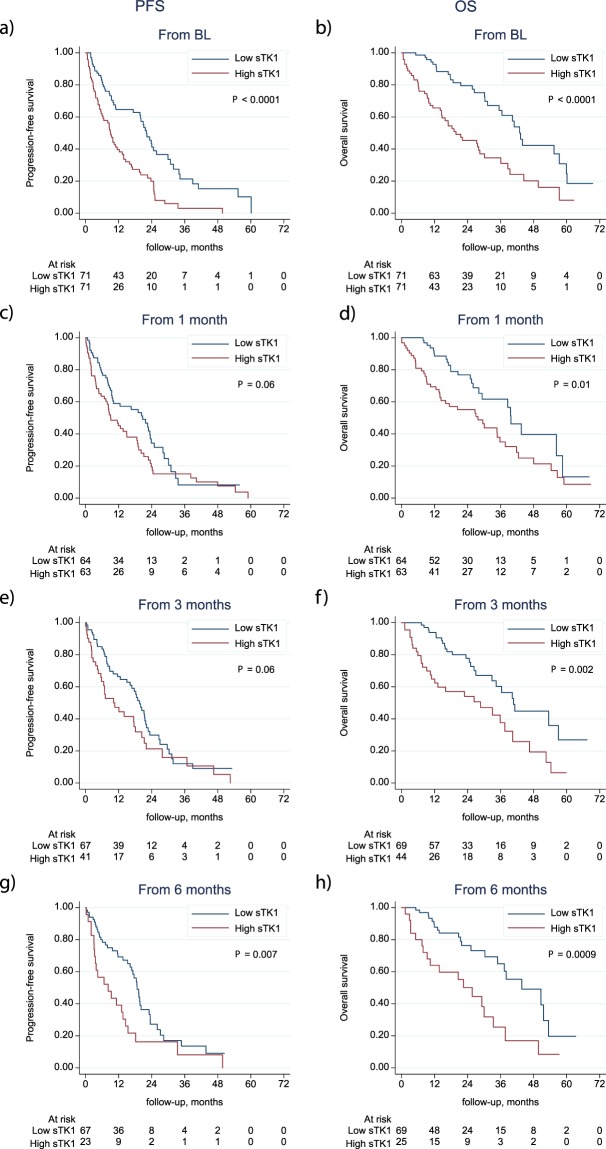
Progression-free and overall survival in relation to sTK1 activity levels. Kaplan-Meier curves displaying PFS and OS in patients with high versus low sTK1 activity levels based on the median sTK1 level cut-off at baseline (**a**,**b**), at 1 month (**c**,**d**), at 3 months (**e**,**f**) and at 6 months (**g**,**h**) during the first 6 months of systemic therapy for MBC. Analyses at 1, 3, and 6 months were performed using landmark analysis, in which the follow-up time was recalculated with a new starting date from the 1, 3, and 6-month sample date, respectively.

The relevance of a median cut-off was further analyzed using quartiles of sTK1 levels. A potential dose-response relationship between sTK1 levels and PFS and OS, respectively, was investigated by dividing the activity levels into quartiles. For both endpoints, Kaplan-Meier curves were similar for the two groups below the median as well as for the two groups above the median, suggesting that the median cut-off was relevant for further survival analyses (Fig. S1). When comparing patients with high (above median) versus low (below median) sTK1 levels at BL, high sTK1 levels were associated with worse PFS (HR: 2.34, CI:1.38–3.45, *P* < 0.001) and OS (HR: 2.58, CI: 1.62–4.13, p < 0.001) (Table 2) as illustrated in Kaplan Meier survival curves (Fig. 3). After adjusting for other known prognostic factors in MBC (age, performance status, grade, subtype, metastasis-free interval, number of metastatic loci and visceral metastases) in a multivariable Cox regression analysis, sTK1 levels at BL were still significant for both PFS (HR: 2.55, CI: 1.53–4.24, *P* < 0.001) and OS (HR: 2.22, CI: 1.22–4.04, *P* = 0.009) (Table 2). During treatment, high sTK1 levels were significantly associated with worse OS from all follow-up time points (1, 3 and 6 months) and with worse PFS from 3 and 6 months (Fig. 3c–h; Table 2). When adjusting for other prognostic variables in multivariable Cox regression analysis there was still a significant prognostic impact of sTK1 levels on OS at 3 and 6 months (Table 2). Due to the wide range of sTK1 levels at BL, we further log transformed sTK1 values and studied associations to PFS and OS on this multiplicative scale. Cox regression analyses showed an impaired PFS (HR: 1.58; CI: 1.24–2.02; *P* < 0.001) and OS (HR: 1.83; CI: 1.35–2.47; *P* < 0.001) per 10-fold increase in sTK1 levels. After adjusting for other prognostic factors in multivariable analyses the hazard ratio for PFS was 2.27 (CI: 1.56–3.30; *P* < 0.001) and for OS 2.97 (CI: 1.77–4.97; *P* < 0.001), respectively (Table S2).

**Table 2:** Hazard ratios for high (>median) versus low (<median) sTK1 activity levels at baseline and during follow-up for 1, 3 and 6 months, estimated by Cox regression analysis.

Time point		PFS			OS		
		HR	CI	*P*-value	HR	CI	*P*-value
**BL**	**UV**	2.34	1.58–3.45	<0.001	2.58	1.62–4.13	<0.001
	**MV**^**a**^	2.55	1.53–4.24	<0.001	2.22	1.22–4.04	0.009
**1 m**	**UV**	1.44	0.96–2.15	0.08	1.69	1.03–2.78	0.04
	**MV**^**a**^	1.43	0.87–2.35	0.16	1.65	0.90–3.04	0.11
**3 m**	**UV**	1.58	1.02–2.44	0.04	2.5	1.51–4.23	<0.001
	**MV**^**a**^	1.68	0.94–3.00	0.08	2.83	1.41–5.68	0.004
**6 m**	**UV**	2.04	1.20–3.48	0.009	3.09	1.72–5.56	<0.001
	**MV**^**a**^	1.74	0.86–3.52	0.125	7.77	2.81–21.49	<0.001

*Abbreviations:* PFS, progression-free survival; OS, overall survival; HR, hazard ratio; CI, confidence interval; UV, univariable analysis; MV, multivariable analysis; BL, baseline; m, months.

^a^Adjusted for age, ECOG (Eastern Cooperative Oncology Group Performance Status), NHG (Nottingham Histological Grade), Subtype, Metastatis-Free Interval, Number of metastatic sites, Site of metastasis (visceral/non-visceral).

### Changes in sTK1 levels for monitoring therapy response

**Figure 4:**
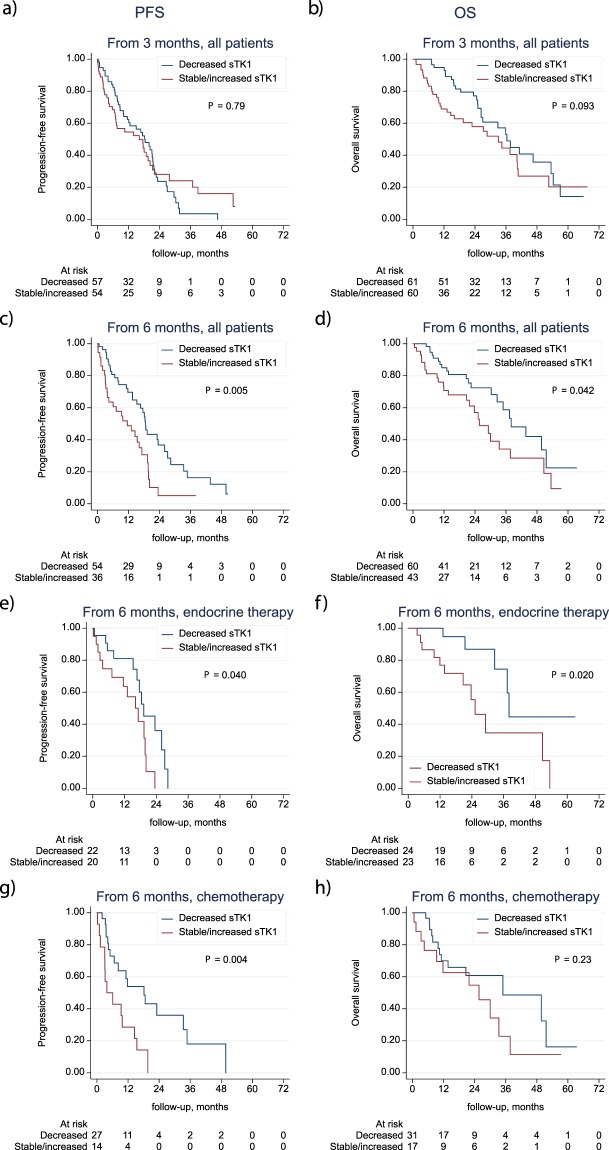
Progression-free and overall survival in relation to changes in sTK1 levels. Kaplan-Meier curves displaying PFS and OS in all patients with decreasing sTK1 levels versus unchanged/increasing sTK1 levels during therapy from baseline to 3 months (**a**,**b**) and from 3 to 6 months (**c**,**d**); and in subgroup analysis from 3–6 months based on treatment modality; endocrine therapy (**e**,**f**) or chemotherapy (**g**,**h**).

To evaluate sTK1 level as a marker of therapy response we analyzed changes in sTK1 levels and correlations to survival (PFS and OS). We applied the cut-off suggested by Malorni *et al*. in the EFECT-study^17^ comparing decreases of>10% to increasing or unchanged levels. We found that in patients with more than a 10% decrease in sTK1 levels from BL to 3 months, there was a trend towards an improved OS (*P* = 0.09) compared to patients with increasing/unchanged sTK1 levels (Fig. 4b). Regarding PFS there was no significant difference between the groups (Fig. 4a). When analyzing even earlier changes from BL to 1 month, there were no significant differences in survival between the two groups (data not shown). However, when analyzing changes in sTK1 levels during further follow-up from 3 to 6 months, there was a significantly improved survival (PFS and OS) in the group of patients with decreasing sTK1 levels compared to patients with increased/unchanged levels (Fig. 4c,d). PFS from 6 months in patients with decreasing sTK1 levels from 3–6 months, was improved with a HR of 0.48 (CI: 0.29–0.81; *P* = 0.005), compared to patients with increasing/unchanged sTK1 levels from 3–6 months. Corresponding HR for OS from 6 months was 0.55 (CI: 0.31–0.99, *P* = 0.04) for patients with decreasing sTK1 levels versus patients with increased/unchanged sTK1 levels. Subgroup analyses based on treatment modality (ET, ChT or HER-2 directed therapy) showed that the improved survival in patients with decreasing sTK1 levels from 3–6 months was significant for both PFS (*P* = 0.04) and OS (*P* = 0.02) in patients receiving ET (Fig. 4e,f). Also for patients treated with ChT, a correlation between decreasing sTK1 levels from 3–6 months, and improved PFS was found (*P* = 0.004) but for OS the difference was not significant (*P* = 0.23; Fig. 4g,h). The subgroup of patients receiving HER2-directed therapy was too small to allow any survival analyses (n = 12).

When further analyzing relative changes in sTK1 levels from BL to 3 months, using log-transformed sTK1 levels, we found no significant correlations to PFS or OS (data not shown). But, when analyzing relative changes from 3 to 6 months, we found that decreasing sTK1 levels correlated to improved PFS and OS (Table S3). Repeated analyses, adjusted for the sTK1 level at three months, in subgroups based on treatment modality showed an improved PFS (HR: 0.24; CI: 0.07–0.82; *P* = 0.023) and OS (HR: 0.18; CI: 0.06–0.51; *P* = 0.001) with decreasing sTK1 levels (Table S3) in patients treated with ET. For ChT treated patients there was also an improved PFS (HR: 0.37; CI: 0.14–0.99; *P* = 0.049) in patients with decreasing sTK1 levels from 3–6 months whereas there was no significant difference in OS (HR: 0.54;CI: 0.21–1.39; *P* = 0.20) (Table S3). The estimated HRs above correspond to a 10-fold decrease in sTK1 levels.

To further evaluate changes in sTK1 activity levels over time between the different treatment groups, we analyzed median levels of sTK1 activity in subgroups of patients based on progression (at evaluations at 3 and 6 months) versus non-progression. We found that sTK1 levels were initially reduced (at 1 month) in ET treated patients irrespective of therapy response (Table S4). However at follow-up time points at 3 and 6 months, increasing sTK1 levels were observed in patients with progression whereas patients with non-progression had continuously reduced sTK1 activity levels at all time-points (Table S4). When analyzing patients treated with ChT, sTK1 levels were initially increased (at 1 month) in patients irrespective of therapy response. However, at longer follow-up time points (at 3 and 6 months) sTK1 levels were continuously increased in patients with progression whereas sTK1 levels in patients with non-progression started to decrease from 3–6 months (Table S4). Notably, patients with early progression also had higher median sTK1 activity levels at BL, where the median sTK1 levels in patients with PD (36 patients) was 560 Du/L (11–27230 Du/L) compared to 227 Du/L (12–35520 Du/L) in patients with non-PD (96 patients) (Table S4).

## Discussion

In the present study, we show that sTK1 activity level (measured with DiviTum^®^), is prognostic for survival (PFS and OS) in patients with newly diagnosed MBC. Further, sTK1 is prognostic for survival during therapy by landmark analyses up to 6 months. Also, decreases in sTK1 levels from 3 to 6 months correlate to improved survival (PFS and OS). Taken together these results suggest that sTK1 evaluation may be clinically useful in MBC patients to improve prognostication and therapy monitoring.

High sTK1 levels correlated to high number of metastatic loci and high CTC number, suggesting sTK1 as a marker reflecting tumor burden. Since TK1 activity has been proposed as a liquid-based proliferation marker it may also be a marker for highly proliferative tumors, hence more aggressive disease^10,19^. In our study there was a borderline significant correlation between high Ki67 expression in the metastasis and high sTK1 levels (*P* = 0.06) supporting this suggestion. Regarding survival, high sTK1 level was an independent prognostic factor for both PFS and OS in patients with newly diagnosed MBC. These results support previous studies reporting impaired survival in patients with high TK1 levels in different malignancies^11–13^. When evaluating sTK1 levels during therapy as a tool for monitoring treatment in MBC we found that the median cut-off of initial sTK1 level was relevant for correlation to both PFS and OS at follow-up time points at 3 and 6 months. However, when further analyzing changes in sTK1 levels over time during treatment we found that decreasing levels from BL to 3 months did not significantly correlate to survival (PFS and OS), although there was a trend towards improved survival (OS) for patients with decreased levels at 3 months compared to patients with increased/unchanged levels. Moreover, when evaluating sTK1 levels at longer follow-up (3–6 months), decreasing sTK1 levels were predictive of improved PFS and OS in the whole cohort. Additionally, subgroup analysis based on treatment modality revealed differences in sTK1 activity patterns over time in ChT treated patients compared to patients receiving ET. Notably, initial sTK1 levels were higher in patients planned to receive ChT compared to ET, which might be explained by the finding that high sTK1 correlates to more metastatic loci, worse performance status, high CTC count and hence more aggressive disease at diagnosis. Further, kinetics of sTK1 activity differed in the different treatment groups, suggesting that ET and ChT have various impact on sTK activity in blood. However, subanalyses of ChT treated patients based on subtype did not reveal any significant differences in median sTK1 levels at baseline between patients with ER positive disease versus TNBC.

In ChT treated patients, we found increasing sTK1 levels observed during the first month of treatment regardless of tumor response, which might reflect a “spike” similar to that reported for CA15–3 in other studies^6,20,21^. However, the mechanisms behind this “spike” phenomena might differ for TK1 compared to CA15–3. TK1 activity may be affected by different chemotherapy agents by interference of deoxy-thymidine-mono-phosphate synthesis resulting in a compensative increase of TK1 activity^22,23^, but also due to increased bone marrow cell proliferation^12^. Also, cell death of malignant proliferative cells can result in release of intracellular TK1 and thereby increased TK1 activity in serum^24^. Hence, early changes in sTK1 levels in previously untreated MBC patients might reflect other mechanisms than tumor proliferation but importantly, over time, the sTK1 levels become more stable and therefore more reliable as a marker of treatment efficacy. When analyzing changes at later time points, a decrease in sTK1 level from 3–6 months was significantly associated with improved OS.

For patients treated with ET in this study, sTK1 levels decreased already at one month and continued to decrease up to 6 months during treatment. When evaluating changes at longer follow-up we found that decreasing sTK1 levels from 3–6 months correlated significantly to improved survival (PFS and OS). This is in line with previous studies reporting that decreasing TK1 activity levels are predictive of prognosis in patients with MBC^14,16,17^. Bonechi *et al*. reported an improved PFS in patients with HR+/HER2- MBC showing decreasing TK1 activity levels during ET^14^. That study only included 31 patients where 10 patients had received previous treatment for MBC and hence cannot be directly compared to our present study. TK1 activity levels in serum have also been evaluated in 242 women in the EFECT trial where women with MBC were treated with fulvestrant or exemestane after progression on nonsteroidal aromatase inhibitors^16,17,25^. Recently published results demonstrated a significant correlation between changes in TK1 activity levels and time to progression^16^. In comparison to our study, patients in the EFECT trial had previously received ET for MBC and the TK1 activity levels were lower at baseline than in our study cohort (median: 97 Du/L versus 204 Du/L). The correlation we report between improved PFS and OS in patients during ET and reduced sTK1 levels after 3 to 6 months of therapy, supports the previously reported findings that sTK1 levels can be used to assess therapy efficacy in MBC at these time points. Our data also suggest that it might be used in patients treated with ChT, however the initial “spike” observed at the first months of therapy can be considered not as a marker of tumor progression but rather a compensatory response that ceases over time with continuation of ChT. Hence, also in ChT treated patients sTK1 levels might be useful for disease monitoring at longer follow-up time points. Compared to other previously published studies evaluating sTK1 in MBC^14,16,17^, our study only included patients previously untreated for MBC. The median sTK1 level at BL was higher in this cohort, suggesting that sTK1 levels are higher in untreated patients, which is also consistent with the results that median sTK1 levels evaluated in the whole population decreased during treatment. However, therapy modalities impact sTK1 levels in different ways which is important to evaluate when considering sTK1 as a potential prognostic and therapy monitoring tool. To further investigate sTK1 levels and different kinetics during therapy, patients with newly diagnosed MBC included in the ongoing observational study SCAN-B-rec (ClinicalTrials.gov NCT03758976) will serve as a validation cohort for sTK1 evaluation.

This is, to our knowledge, the largest study of serial sTK1 level evaluation in women with newly diagnosed MBC (patients previously untreated for MBC). A limitation of this study is that since it included MBC patients irrespective of treatment regimen and sTK1 levels seem to be influenced differently by diverse therapies, sub-group analysis based on therapy modality may be underpowered to detect significant correlations to survival. Further, the sTK1-analyses were performed retrospectively and serum samples were not available for all patients. However, we could demonstrate sTK1 level as an independent prognostic factor in patients with newly diagnosed MBC. Also, decreasing sTK1 levels at 6 months follow-up during therapy was associated with extended survival. To conclude, these results suggest that sTK1 may be clinically useful in prognostication and monitoring of therapy in MBC patients, but to further explore in what settings sTK1 is most useful, future studies are needed.

## Methods

### Patients and study design

156 MBC patients planned to start first-line systemic therapy were included in a prospective monitoring trial (ClinicalTrials.gov NCT01322893) at Skåne University Hospital and Halmstad County Hospital, Sweden, from 2011–2016 and clinical data has been reported^26^. Briefly, inclusion criteria were MBC diagnosis, age ≥18 years, Eastern Cooperative Oncology Group (ECOG) performance status score 0–2, and predicted life expectancy of >2 months. Initially 168 patients were included, 12 patients were excluded when reviewing patient records after study completion; 8 patients did not meet inclusion criteria and 4 patients were found to have only locoregional recurrence. After study inclusion, the participating patients were planned to start first-line systemic therapy for MBC according to national guidelines. Patient, tumor and treatment data were prospectively collected in case report forms (CRF). Clinical evaluation, including imaging, was performed approximately every 3 months or at the discretion of the treating physician. Modified Response Evaluation Criteria In Solid Tumors (RECIST) 1.1^5^ was used to define progression (Progressive Disease; PD) versus non-progression (stable disease; SD, partial reponse; PR or complete response; CR). Serum samples were collected at BL for 142 patients, and after 1, 3, and 6 months of treatment. Twelve patients at one center (Halmstad County Hospital) and two other patients (one patient in Lund and one patient in Malmö) did not have serum samples collected due to logistic issues. Circulating tumor cells (CTCs) were enumerated using the CellSearch™ system, described in detail previously^26^. Preliminary results have been reported at a poster session at SABCS (San Antonio Breast Cancer Symposium) 2017^27^.

### TK1 activity level analysis

Serum TK1 activity levels were determined using the DiviTum^®^ assay (Biovica, Sweden) in accordance with the manufacturer’s instructions, which has previously been reported^18^. Briefly, serum was mixed with reaction mixture in a 96-well enzyme-linked immunosorbent assay (ELISA), bromodeoxyuridine (BrdU) monophosphate was generated by TK reaction, phosphorylated to BrdU triphosphate and incorporated into a synthetic DNA strand. An anti-BrdU monoclonal antibody conjugated to enzyme alkaline phosphatase and a chromogenic substrate was used to detect BrdU incorporation. The absorbance readings were converted using standards with known TK activity (working range from 20 to 4000 Du/L). Biovica (Uppsala, Sweden) performed all sTK1 analyses, and the company was blinded to patient and tumor data.

### Statistical analysis

Pearson’s chi-squared test or Fisher’s exact test were used for comparison of categorical or categorized patient and tumor characteristics. Pearson’s chi-squared test for trend was used for comparison of ordinal variables and Mann-Whitney U-test (two categories) or Kruskal-Wallis test (more than two categories) were used when comparing variables measured on a continuous scale. The evidence for change in sTK1 activity levels between two time points was evaluated with Wilcoxon matched-pairs signed-ranks test.

This study was performed in accordance with REMARK criteria (REporting recommendations for tumor MARKer)^28,29^. Time to progression or death from any cause was calculated from the respective serum sampling date. If an outcome was not yet reached, the corresponding time variable was censored at last follow-up. To illustrate survival, we used Kaplan-Meier plots and log-rank tests were used to compare survival between subgroups. Landmark analyses were performed for variables measured at 1, 3, and 6 months - i.e by redefining time zero in the survival analyses. Cox proportional hazards regression was used to estimate uni- and multivariable hazard ratios (HRs) for PFS and OS. Due to the extreme range of sTK1 levels at baseline, sTK1 levels were log-transformed with base 10 for some analyses. Hazard ratios corresponding to a factor 10 change in sTK1 between two time points were estimated with and without adjustment for the sTK1 level at the first of the two time points. Proportional hazards assumptions were checked graphically. The statistics packages IBM SPSS Statistics (version 24.0, IBM, Armonk, NY, USA) and Stata 15.1 (StataCorp LLC, College Station, TX, USA) were used.

### Ethical approval

This study was approved by Lund University Ethics Committee (LU 2010/135). All procedures performed in studies involving human participants were in accordance with the ethical standards of the institutional research committee (Lund University Ethics Committee) and with the 1964 Helsinki declaration and its later amendments or comparable ethical standards.

### Informed consent

Informed consent was obtained from all individual participants included in the study.

## Supplementary information

Supplementary information.

## Data Availability

The datasets analyzed during the current study are available from the corresponding author on reasonable request.
