# Supplementary material for: Serial evaluation of serum thymidine kinase activity is prognostic in women with newly diagnosed metastatic breast cancer
Source: Sci Rep. 2020 Mar 11;10:4484. doi: 10.1038/s41598-020-61416-1 (PMC7066186; doi:10.1038/s41598-020-61416-1)

Supplementary Information

Scientific Reports

Serial evaluation of serum thymidine kinase activity is prognostic in women with newly diagnosed metastatic breast cancer

Authors: Anna-Maria Larsson*[1,2], Pär-Ola Bendahl[1], Kristina Aaltonen[3], Sara Jansson[1], Carina Forsare[1], Mattias Bergqvist[4] , Charlotte Levin Tykjaer Jorgensen[1] , Lisa Rydén[5,6]

Affiliations: [1]Department of Clinical Sciences, Lund, Division of Oncology and Pathology, Lund University, Lund, Sweden; [2]Department of Hematology, Oncology and Radiation Physics, Skåne University Hospital, Lund, Sweden; [3]Department of Laboratory Medicine, Division of Translational Cancer Research, Lund University, Lund, Sweden; [4]Biovica, Uppsala, Sweden; [5] Department of Clinical Sciences, Lund, Division of Surgery, Lund University, Lund, Sweden; [6]Department of Surgery, Skåne University Hospital, Lund, Sweden

***Corresponding author:** Anna-Maria Larsson, MD, PhD,

Address: Department of Clinical Sciences, Lund, Division of Oncology and Pathology, Medicon Village, Building 404, Lund University, SE-22381 Lund, Sweden

Email: anna-maria.larsson@med.lu.se

Supplementary Table S1. Median sTK1 activity levels at baseline and during 1, 3 and 6 months of therapy in all patients and in subgroups based on treatment modality

**Treatment group**		**Baseline Median TK1 level, Du/L (range)**	**1 month Median TK1 Level, Du/L (range)**	**3 months Median TK1 Level, Du/L (range)**	**6 months Median TK1 Level, Du/L (range)**
**All (n=134)**		391 (11-35520)	433 (9-40440)	200 (5-38310)	131 (4-28030)
**Chemotherapy (n=64)**		420 (12-35520)	874 (21-34510)	759 (20-38310)	387 (14-28030)
**Subtype in Chemotherapy treated**	**ER+ (n=41)**	423 (14-35520)	934 (21-34510)	854 (20-38310)	392 (14-28030)
	**TNBC (n=22)**	435 (12-3379)	804 (92-7660)	646 (87-3476)	283 (33-18360)
**Endocrine therapy (n=57)**		204 (11-27230)	93 (9-31170)	79 (5-17720)	55 (4-4480)
**HER2 directed therapy (n=13)**		1037 (16-22740)	913 (47-40440)	197 (47-3709)	107 (21-2523)

Abbreviations: Du/L, Divitum units per Litre; ER+, estrogen receptor positive; TNBC, triple negative breast cancer

Supplementary Table S2. Cox regression analyses of hazard ratios in relation to log10 sTK1 levels in univariable and multivariable models

		**PFS**			**OS**		
		**HR**	**CI**	***P*-value**	**HR**	**CI**	***P*-value**
**Baseline**	**UV**	1.58	1.24-2.02	<0.001	1.83	1.35-2.47	<0.001
	**MV[a]**	2.27	1.56-3.30	<0.001	2.97	1.77-4.97	<0.001

PFS, progression-free survival; OS, overall survival; HR, hazard ration; CI, confidence interval; UV, univariable analysis; MV, multivariable analysis

[a]Adjusted for age, ECOG (Eastern Cooperative Oncology Group Performance Status), NHG (Nottingham Histological Grade), Subtype, Metastatis-Free Interval, Number of metastatic sites, Site of metastasis (visceral/non-visceral

Supplementary Table S3. Cox regression analysis evaluating hazard ratios in patients with Log10 changes (decrease versus increase/unchanged) in sTK1 levels from 3 to 6 months, adjusted for sTK1 level at three months and subgroup analyses based on treatment modality (ET versus ChT)

**Treatment type**	**PFS**			**OS**		
	**HR**	**CI**	***P*-value**	**HR**	**CI**	***P*-value**
**All**	0.43	0.20-0.90	0.026	0.34	0.16-0.72	0.004
**ET**	0.24	0.07-0.82	0.023	0.18	0.06-0.51	0.001
**ChT**	0.37	0.14-0.99	0.049	0.54	0.21-1.39	0.20

PFS, progression-free survival; OS, overall survival; HR, hazard ration; CI, confidence interval; UV, univariable analysis; MV, multivariable analysis; ET, endocrine therapy; ChT, chemotherapy

Supplementary Table S4. Median sTK1 activity levels at baseline and during 1, 3 and 6 months of therapy based on treatment modality and response evaluation (at 3 and 6 months)

**Treatment type**	**Time point**	**PD**			**Non-PD**		
		**median**	**range**	**n**	**median**	**range**	**n**
**All**	**BL**	560	11-27230	36	227	12-35520	96
	**1m**	1017	12-31170	36	336	9-40440	96
	**3m**	693	18-17720	31	181	5-38310	91
	**6m**	545	27-18360	18	103	4-28030	86
**ET**	**BL**	250	11-29230	14	172	13-2352	43
	**1m**	89	12-31170	14	93	9-1788	43
	**3m**	89	18-17720	12	78	5-2620	41
	**6m**	147	27-2281	8	41	4-4480	39
**CT**	**BL**	718	83-2677	20	374	12-35520	43
	**1m**	1258	83-7660	20	733	21-34510	43
	**3m**	1504	87-3700	18	636	10-38310	42
	**6m**	1145	125-18360	9	356	14-28030	40

PD, Progressive Disease; non-PD, non Progressive Disease, BL, baseline; ET, endocrine therapy; CT, chemotherapy

Supplementary Figure S1. Progression-free and overall survival in patients with MBC based on quartiles of sTK1 levels at baseline

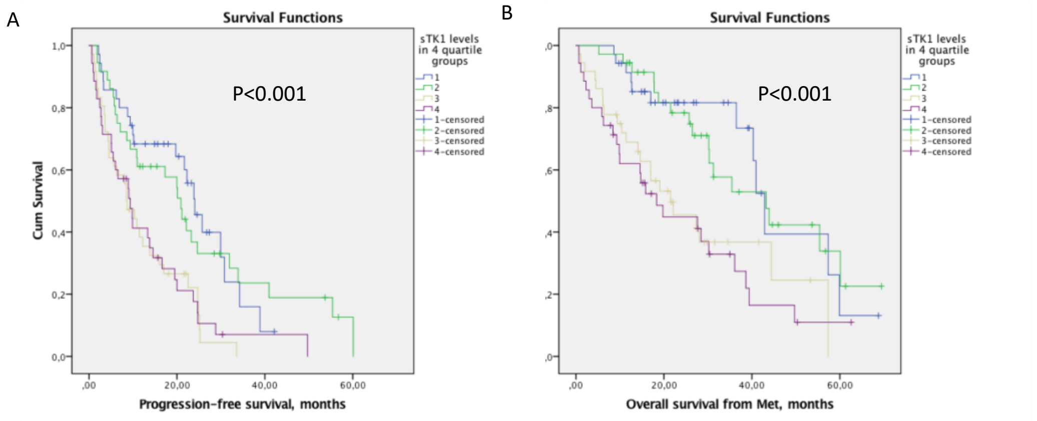

Supplementary Figure S2: Progression-free (A-B) and overall survival (C-D) in relation to changes in sTK1 levels during therapy from baseline to 3 months based on treatment modality: ET (A, C) versus ChT (B, D)

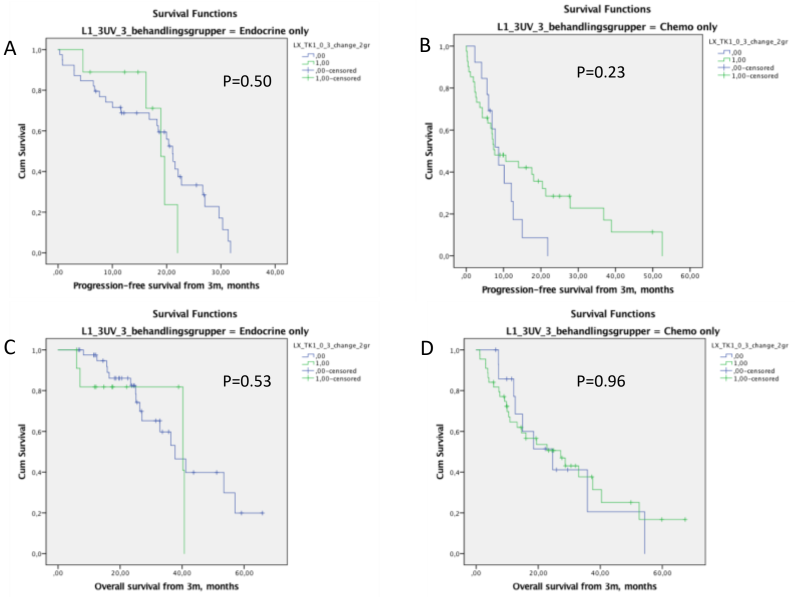

Supplement: Supplementary file 1 — Supplementary information. [file 41598_2020_61416_MOESM1_ESM.pdf]
